# Supplementary material for: Engineered proteins with sensing and activating modules for automated reprogramming of cellular functions
Source: Nat Commun. 2017 Sep 7;8:477. doi: 10.1038/s41467-017-00569-6 (PMC5589908; doi:10.1038/s41467-017-00569-6)
Supplement: Supplementary file 1 — Supplementary Information [file 41467_2017_569_MOESM1_ESM.pdf]

Description of Supplementary Files

Title: Supplementary Information

Description: Supplementary Figures and Supplementary Table

Title: Peer Review File

Title: Supplementary Movie 1

Description: The FRET signals of a MEF cell transfected with the Shp2-iSNAP before and after PDGF stimulation.

Title: Supplementary Movie 2

Description: Activation of SIRPα Shp2-iSNAP by CD47 coated beads. Movie represents FRET/CFP ratiometric (left) and DIC (right) images of RAW264.7 macrophages expressing SIRPα Shp2-iSNAP upon stimulation of CD47&IgG coated beads. Cold and hot colors represented low and high FRET/CFP ratio of iSNAP, respectively.

Title: Supplementary Movie 3

Description: Activation of SIRPα Shp2-iSNAP during phagocytosis of human RBCs by RAW264.7 macrophages. Movie represents FRET/CFP ratiometric (left) and DIC (right) images of RAW264.7 macrophages expressing SIRPα Shp2-iSNAP upon stimulation of opsonized human RBCs. Cold and hot colors represented low and high FRET/CFP ratio of iSNAP, respectively.

Title: Supplementary Movie 4

Description: Activation of SIRPα Shp2-iSNAP during phagocytosis of Toledo by BMDMs. Movie represents FRET/CFP ratiometric (left) and DIC (right) images of BMDMs expressing SIRPα Shp2-iSNAP (upper panels) or SIRPα Shp2-iSNAP ΔPTP (lower panels) with stimulation of opsonized Toledo cells. Cold and hot colors represented low and high FRET/CFP ratio of iSNAP, respectively. It is note that the BMDM expressing SIRPα Shp2-iSNAP presented stronger phagocytic ability comparing to the BMDM expressing SIRPα Shp2-iSNAP mutant (ΔPTP), which is incapable of engulfing Toledo cells.

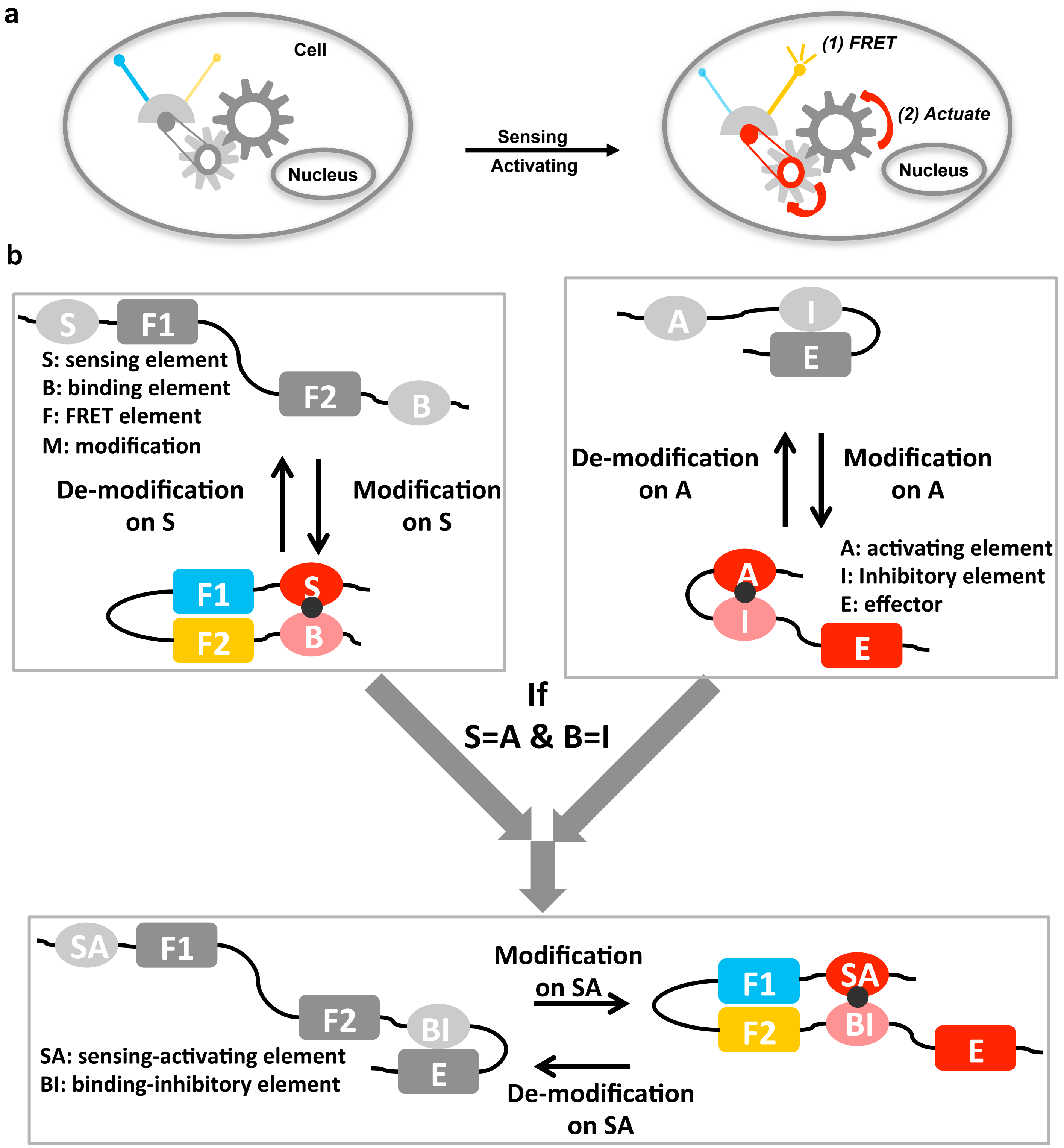

Supplementary Figure 1

(**a**) The concept of *i*SNAPs capable of detecting molecular signals and activating to reprogram cellular functions. (**b**) The general design strategy for *i*SNAPs. A typical biosensor based on FRET consists of a sensing (S) and a binding (B) element, with the sensing element upon stimulation undergoing posttranslational modifications and interactions with the binding element to alter the relative orientation/distance between the conjugated donor and acceptor fluorescent proteins (F1 and F2), leading to detectable FRET changes (Upper left panel). A typical protein activator has an enzymatic domain (E) masked by an inhibitory element (I), which can be relieved by the interaction with an activating element (A) upon stimulation to expose and activate the enzymatic domain (Upper right panel). The combination of these two modules, utilizing the sensing and binding elements of the biosensor to serve as the activating (SA) and inhibitory (BI) elements of the activator, respectively, can provide a generally applicable strategy to engineer an *i*SNAP capable of surveying the intracellular space and taking corresponding actions based on the detected signals (Lower panel).

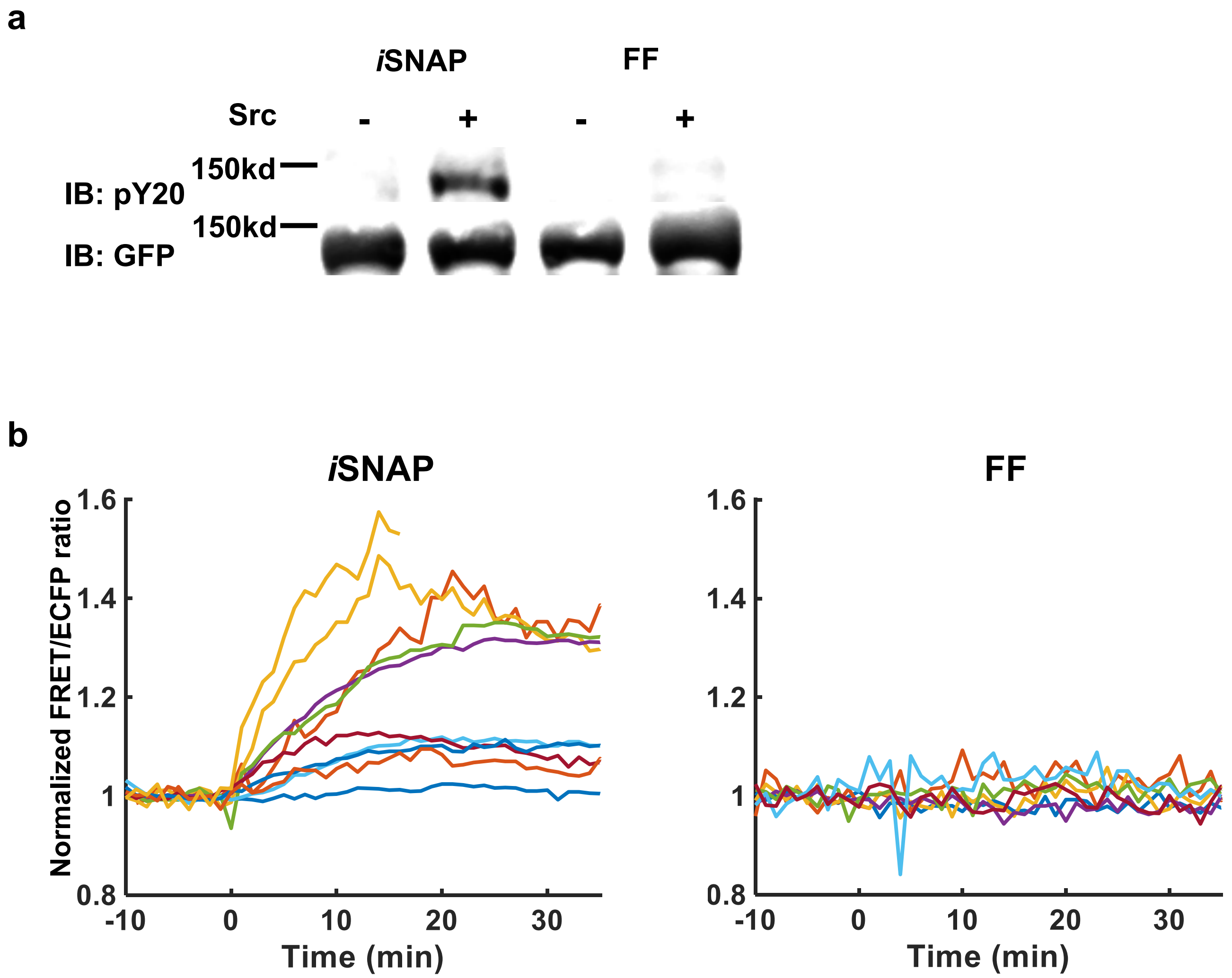

Supplementary Figure 2

(**a**) The tyrosine phosphorylation of the Shp2-*i*SNAP. The phosphorylation levels of the Shp2-*i*SNAP and its FF mutant upon Src incubation *in vitro* were detected by Western blot with anti-phosphotyrosine antibody. (**b**) The ratio time courses of individual MEFs expressing the Shp2-*i*SNAP (left panel, n=10) or its FF mutants (right panel, n= 7).

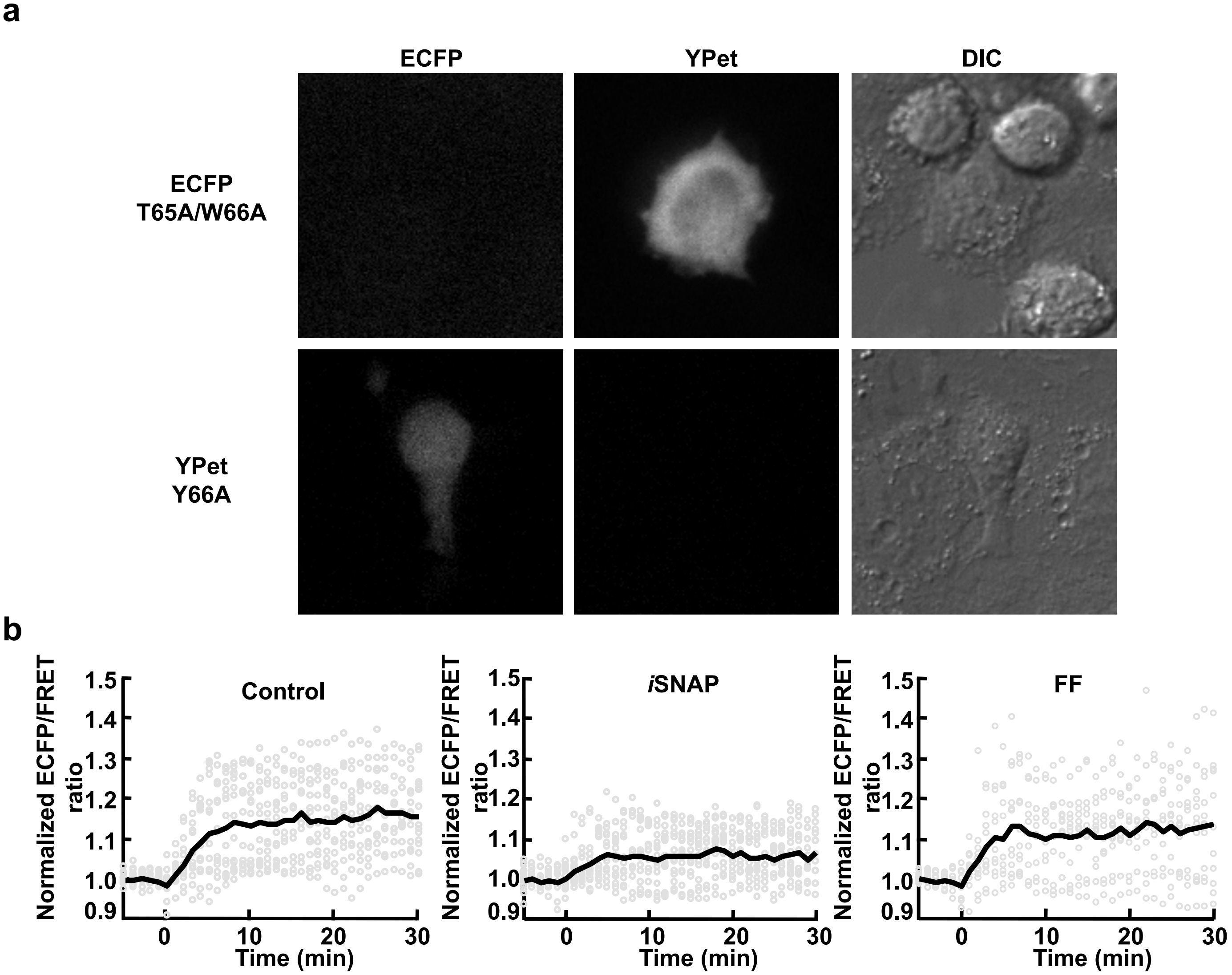

Supplementary Figure 3

Activated Shp2-*i*SNAP suppressed the PDGF-induced increase of FAK activity. (**a**) Images confirmed the mutations in the dark Shp2-*i*SNAP in destroying the fluorescence of specifically targeted fluorescent proteins. (Upper panels) W66A mutation in ECFP specifically eliminated the ECFP fluorescence but not that of YPet. (Lower panels) Y66A mutation in YPet specifically eliminated the YPet fluorescence but not that of ECFP. (**b**) The time courses of emission ratio of the FAK biosensors from different MEFs expressing the control vector (n=15), Shp2-*i*SNAP (n=17) or its FF mutant (n=12), before and after PDGF stimulation.

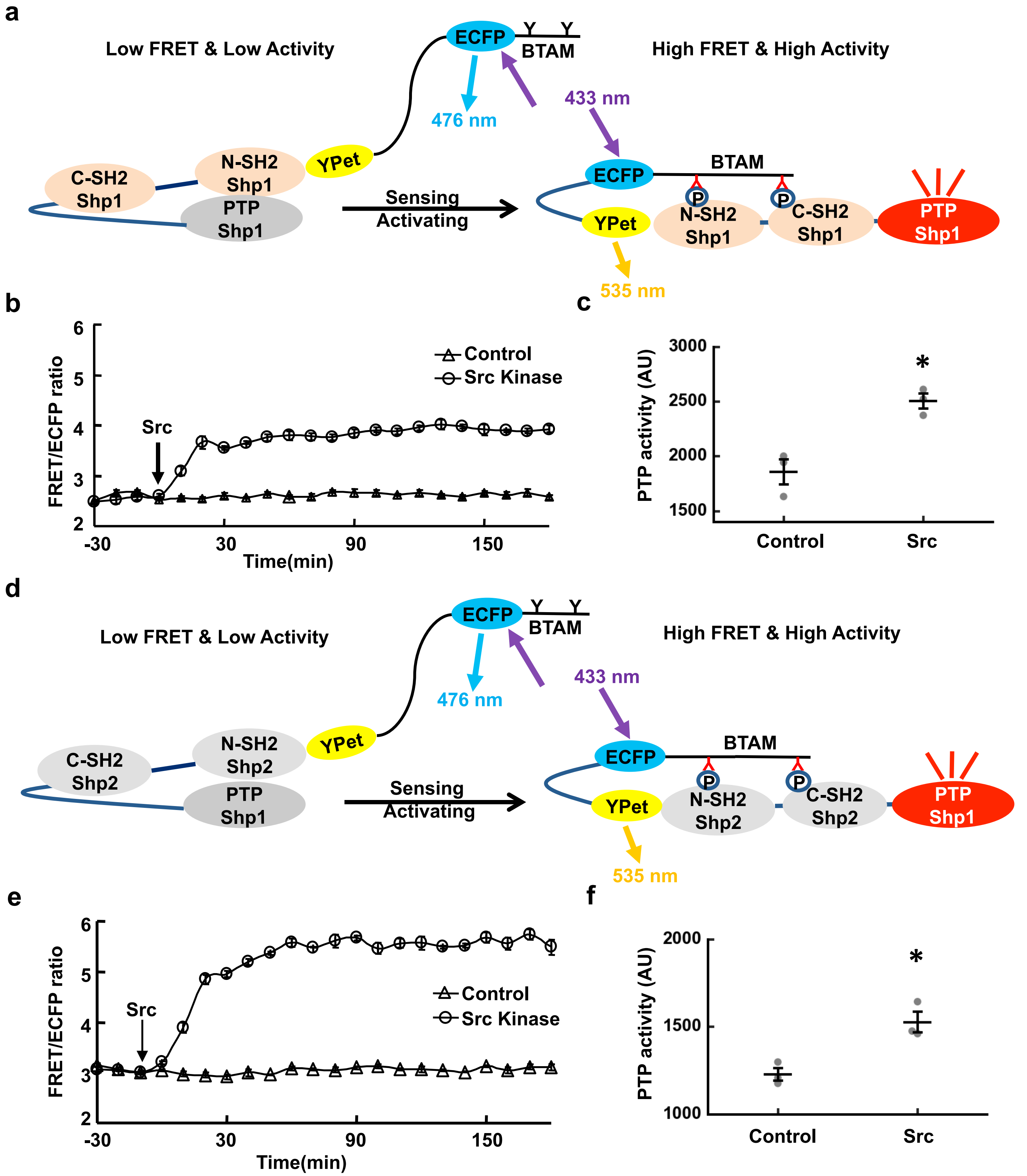

Supplementary Figure 4

The design and characterization of the Shp1 and Shp2/Shp1 hybrid *i*SNAP *in vitro*. (**a**) Schematic drawing of the Shp1-*i*SNAP and its putative activation mechanism, with the two sensing tyrosine sites as indicated. The activated PTP domain is colored in red. (**b**) The emission ratio time courses and (**c**) phosphatase activities of Shp1-*i*SNAP with or without Src incubation *in vitro* (n=3, 3). (**d**) Schematic drawing of the Shp2/Shp1 hybrid *i*SNAP (two SH2 domains are from Shp2 and the PTP domain is from Shp1) and its putative activation mechanism, with the two sensing tyrosine sites as indicated. The activated Shp1 PTP domain is colored in red. (**e**) The emission ratio time courses and (**f**) phosphatase activities of hybrid *i*SNAP with or without Src incubation *in vitro* (n=3, 3). Error bars represent s.e.m. *, $P < 0.05$.

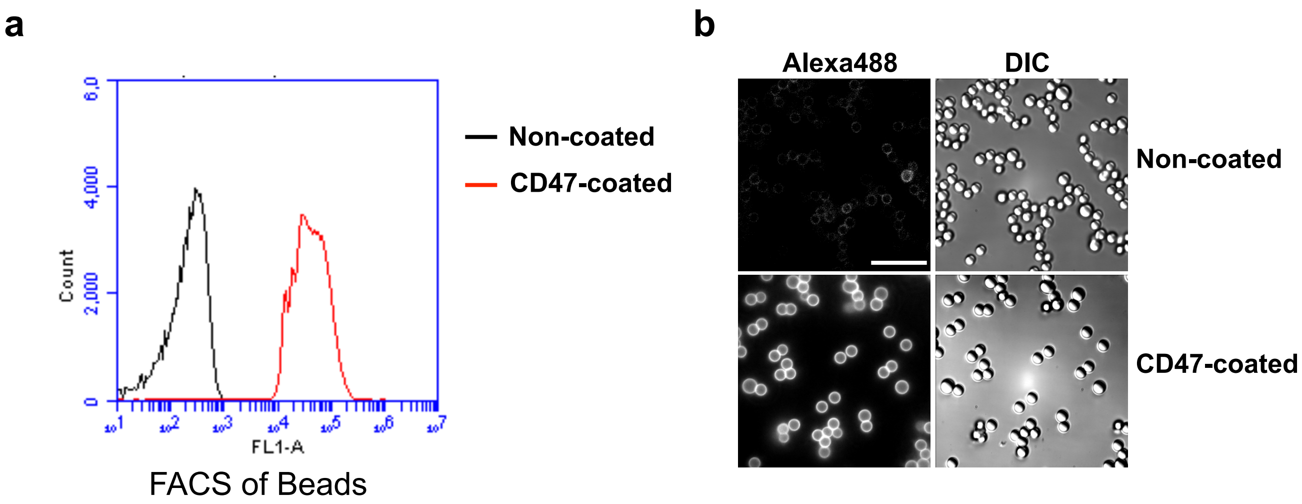

Supplementary Figure 5
Coating of CD47 on polystyrene beads. CD47-coated streptavidin polystyrene beads were stained by CD47 antibody and detected by an Alexa Fluor 488-conjugated secondary antibody using (**a**) flow cytometry and (**b**) fluorescence microscope.

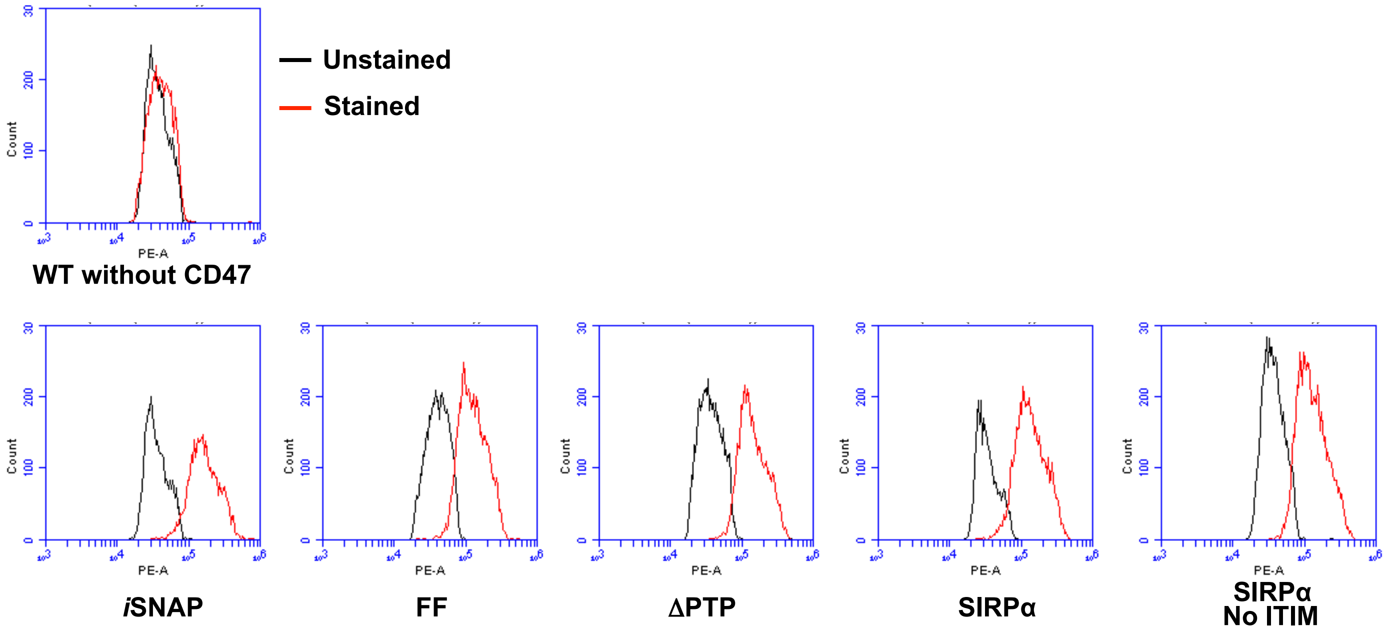

Supplementary Figure 6
Binding of soluble human CD47 to HEK293T cells expressing SIRPα Shp2-*i*SNAP or its control constructs. HEK293T cells transfected with SIRPα Shp2-*i*SNAP or control constructs (FF represents SIRPα Shp2-*i*SNAP with FF mutations in its BTAM peptide; ΔPTP represents SIRPα Shp2-*i*SNAP with the PTP domain truncated; SIRPα represents the full length SIRPα fused with YPet; SIRPα-no ITIM represents the ITIM truncated SIRPα fused with YPet) were incubated with (red) or without (black) soluble biotinylated CD47 and then stained with PE-conjugated streptavidin. Intensity of surfaced bound CD47 was quantified by flow cytometry.

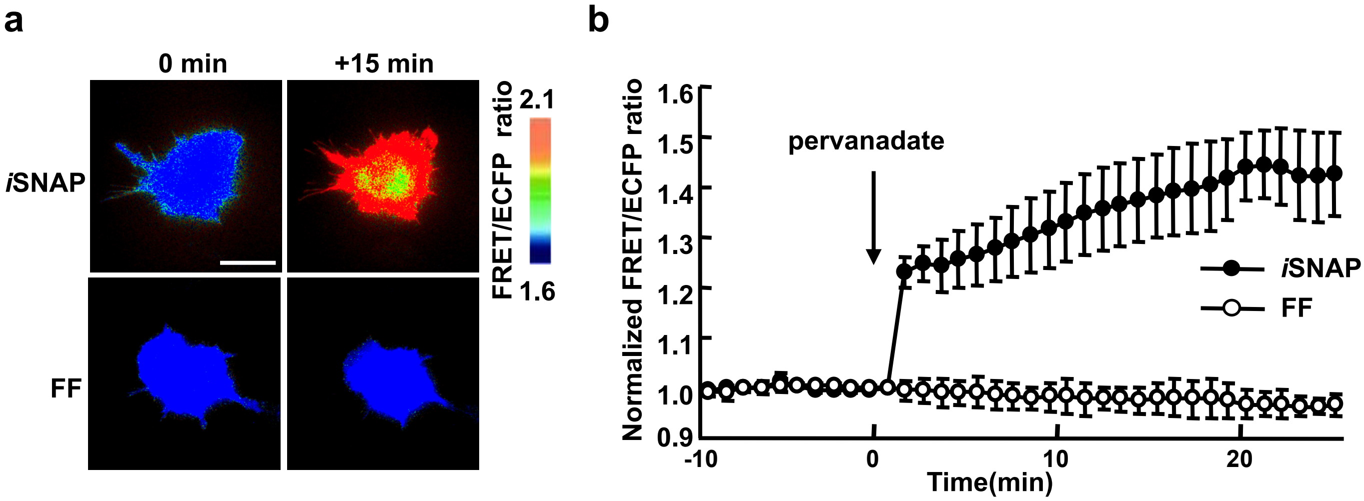

Supplementary Figure 7

The FRET signal of SIRPα Shp2-*i*SNAP is dependent on the phosphorylation of tyrosine in BTAM. (**a**) The ratiometric images or (**b**) the emission ratio time course (mean ± s.e.m.) of RAW264.7 macrophages expressing SIRPα Shp2-*i*SNAP or FF mutants upon 100 μM pervanadate stimulation, respectively. Color scale bar represent the FRET/CFP emission ratio, with cold and hot colors representing low and high FRET efficiency of the *i*SNAP, respectively. Size scale bar, 20 μm.

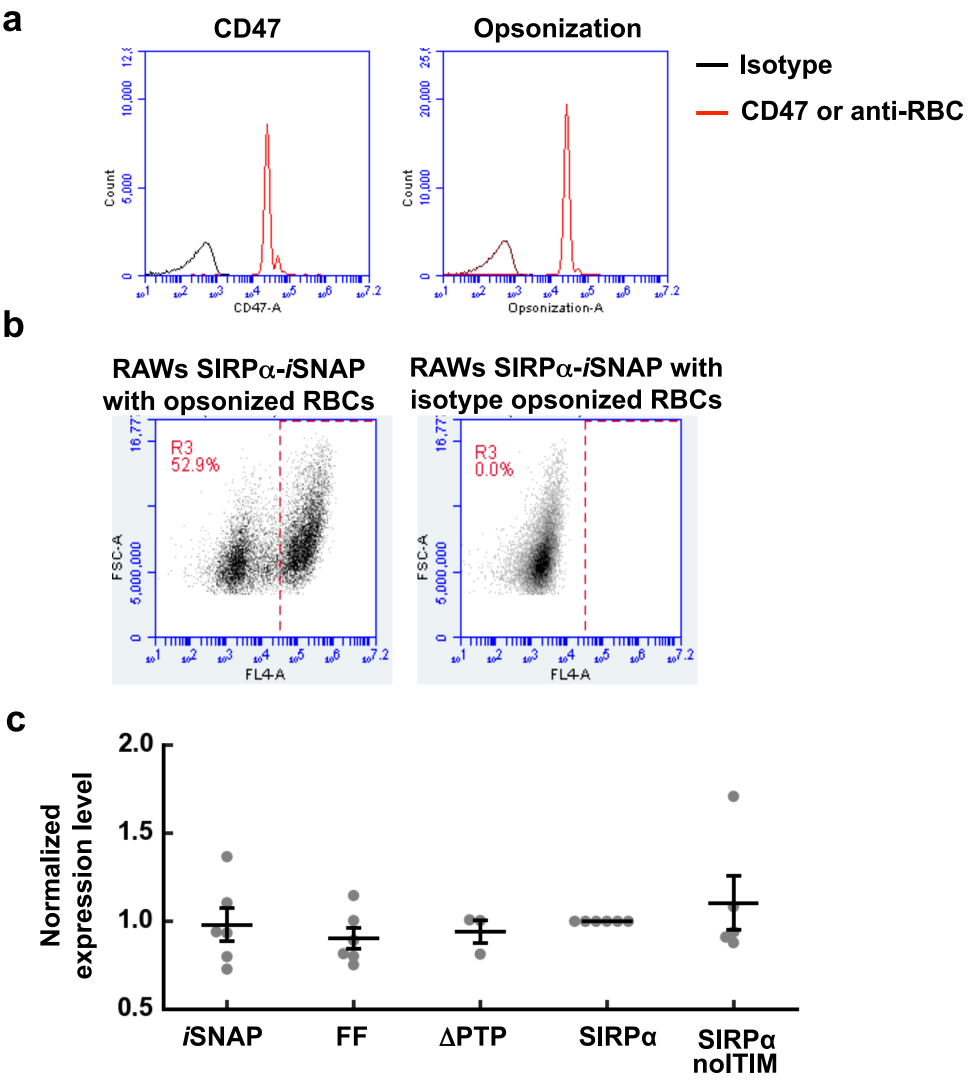

Supplementary Figure 8

Phagocytosis of RBCs by engineered RAW264.7 macrophages. (**a**) Detection of surface CD47 expression and opsonization of RBCs. RBCs were incubated with isotype IgG or mouse anti-human CD47 IgG followed by staining with Alexa Fluor 488-conjugated goat anti-mouse antibody. For opsonization detection (right plots), RBCs were incubated with isotype IgG or rabbit anti-hRBC IgG, followed by staining with Alexa Fluor 594-conjugated goat anti-rabbit IgG secondary antibody. (**b**) Opsonin-dependent phagocytosis of RBCs by engineered RAW264.7 macrophages. RBCs opsonized with anti-human RBC IgG or nonspecific isotype were incubated with RAW264.7 macrophages expressing SIRPα Shp2-*i*SNAP at 37 °C for 30 min. Ingested RBCs was quantified by flow cytometry. Red box indicated the population of macrophages engulfing RBCs. (**c**) Normalized expression level of SIRPα Shp2-*i*SNAP and its control constructs in RAW264.7 cells. Error bars represent s.e.m. (n= 6, 6, 3, 6, 5, 5)

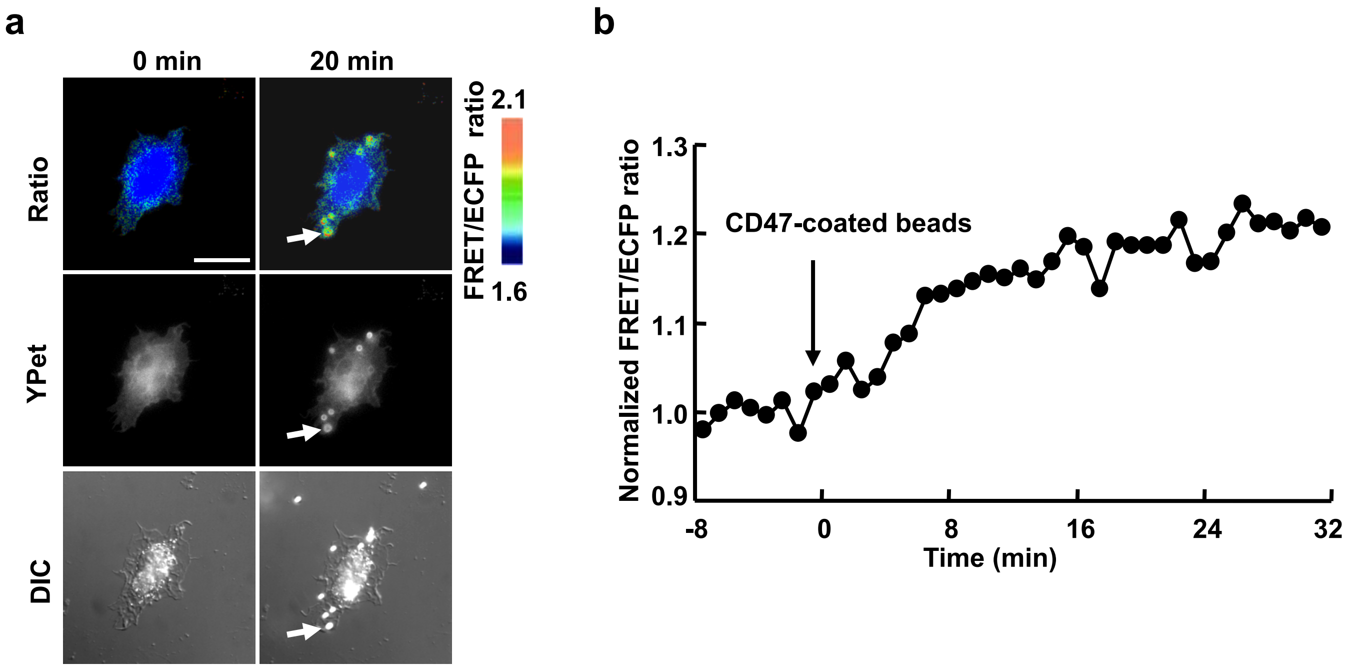

Supplementary Figure 9
CD47 beads caused activation of SIRPα Shp2-*i*SNAP in BMDMs. (**a**) Ratiometric, YPet and DIC images of a BMDM expressing SIRPα Shp2-*i*SNAP before and after incubation with polystyrene beads coated with CD47. (**b**) Time course of FRET/ECFP ratio of SIRPα Shp2-*i*SNAP in response to CD47-coated beads indicated by arrows in (**a**).

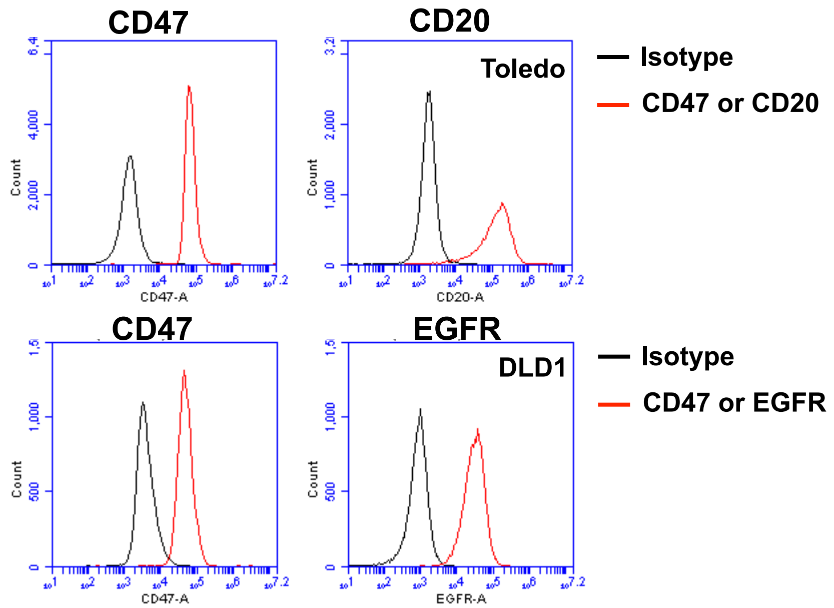

Supplementary Figure 10
Phagocytosis of cancer cells by engineered BMDMs. (**a**) Detection of surface CD47 and CD20 or EGFR expression on Toledo and DLD1 cells. For CD47 staining (left plots), Toledo and DLD1 cells were incubated with isotype IgG or mouse anti-human CD47 IgG followed by staining with Alexa Fluor 488-conjugated goat anti-mouse antibody. For CD20 or EGFR detection (right plots), Toledo and DLD1 cells were incubated with isotype IgG, rituximab (anti-CD20) or cetuximab (anti-EGFR), respectively, followed by staining with PE-conjugated rabbit anti-mouse secondary antibody.

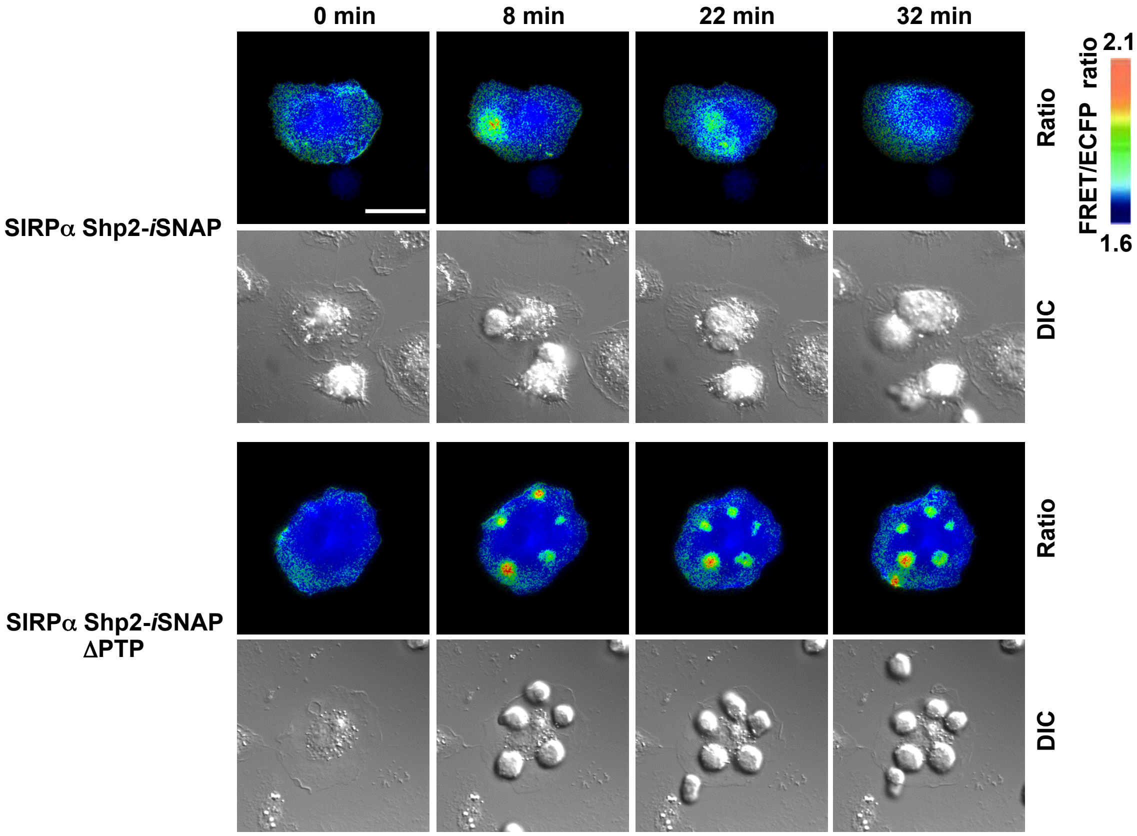

Supplementary Figure 11

Activation of SIRPα Shp2-*i*SNAP during phagocytosis of Toledo by BMDMs as represented in movie 4. Representative FRET/CFP ratiometric (upper) and DIC (lower) images of BMDMs expressing SIRPα Shp2-*i*SNAP (upper panels) or SIRPα Shp2-*i*SNAP ΔPTP (lower panels) before and after the engagement of opsonized Toledo cells. Cold and hot colors represent low and high FRET/CFP ratio of *i*SNAP, respectively. SIRPα Shp2-iSNAP, n=7; SIRPα Shp2-iSNAP ΔPTP, n=5. Scale bar, 20 μm.

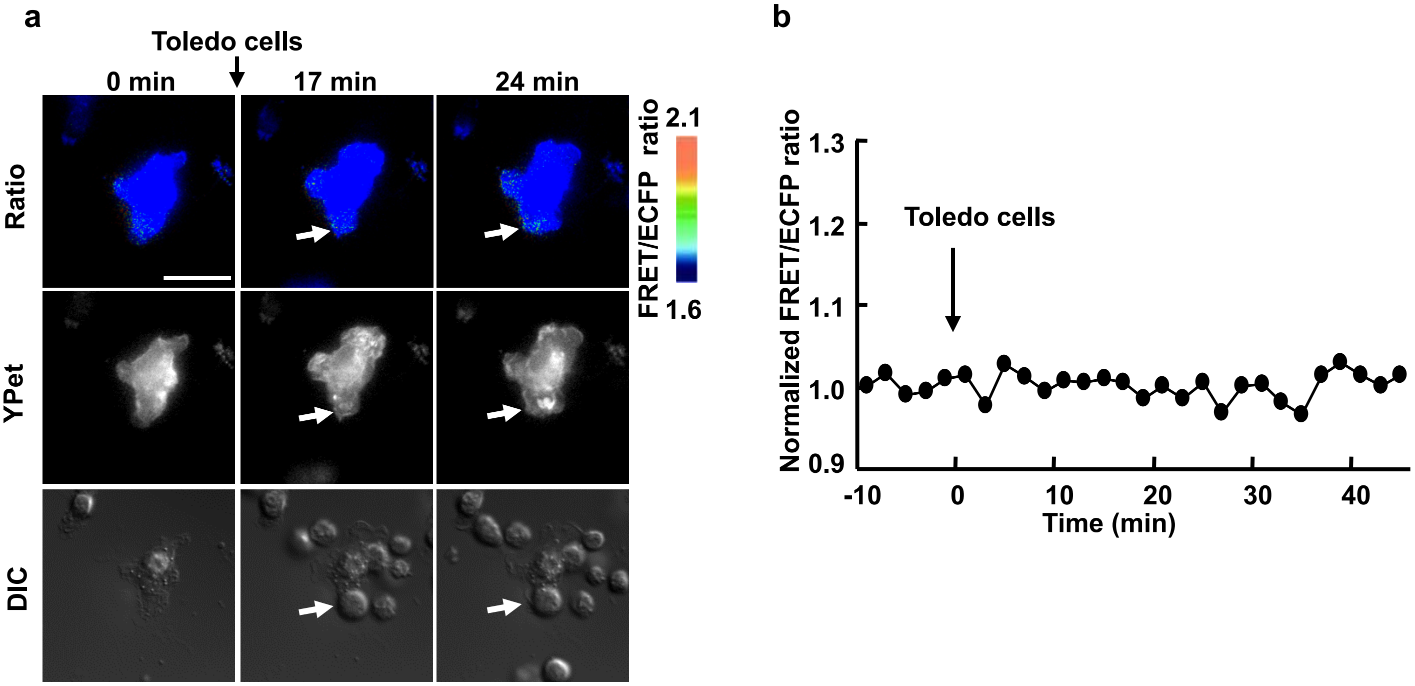

Supplementary Figure 12

Phagocytosis of Toledo cells failed to activate SIRPα Shp2-*i*SNAP FF mutant in BMDMs (**a**) Ratiometric, YPet and DIC images of a BMDM expressing SIRPα Shp2-*i*SNAP FF mutant before and after incubation with rituximab opsonized Toledo cells for various periods of time. (**b**) Time course of FRET/ECFP ratio of SIRPα Shp2-*i*SNAP FF mutant in response to Toledo cells indicated by arrows in (**a**). Color scale bar represent the FRET/CFP emission ratio, with cold and hot colors representing low and high FRET efficiency of the *i*SNAP, respectively. Size scale bar, 20 μm.

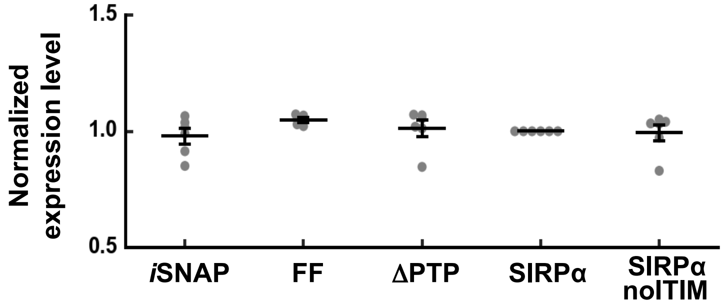

Supplementary Figure 13
Normalized expression level of SIRPα Shp2-*i*SNAP and its control constructs in BMDMs in Toledo cells for phagocytosis assays. Error bars represent s.e.m. (n= 6, 5, 6, 6, 5)

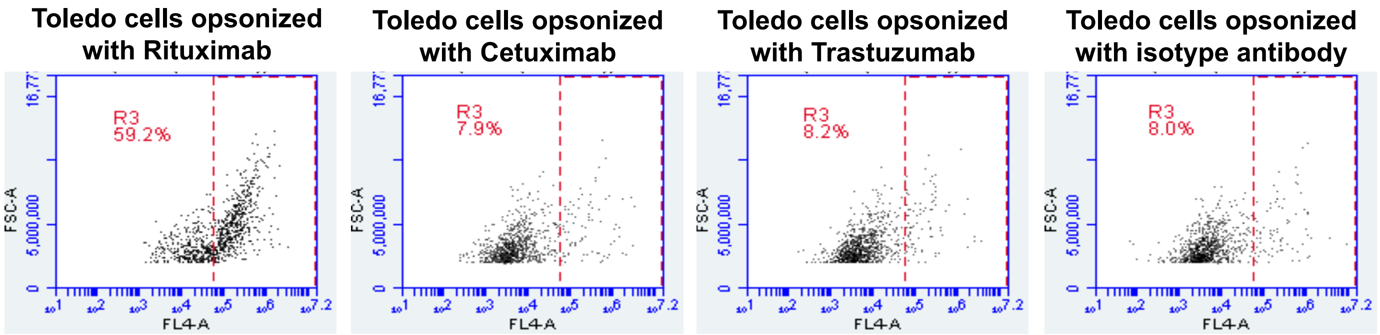

Supplementary Figure 14
Opsonin-dependent phagocytosis of Toledo cells by engineered BMDMs. Toledo cells were added to BMDMs and incubated at 37°C for 4 hours with indicated antibodies (rituximab (10 μg ml^-1), cetuximab (2 μg ml^-1), trastuzumab (2 μg ml^-1), or isotype IgG (2 μg ml^-1)).

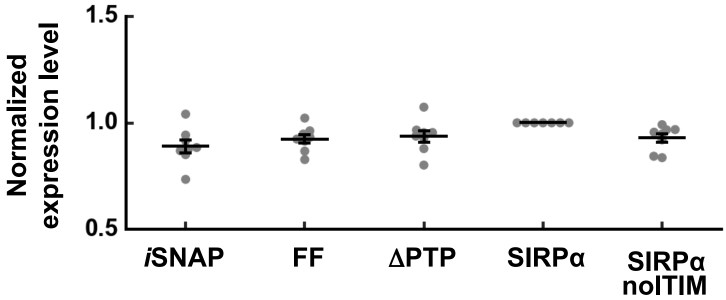

Supplementary Figure 15
Normalized expression level of SIRPa Shp2-*i*SNAP and its control constructs in BMDMs in phagocytosis assay of DLD1 cells. Error bars represent s.e.m. (n= 8, 8, 8, 8, 8, 8)

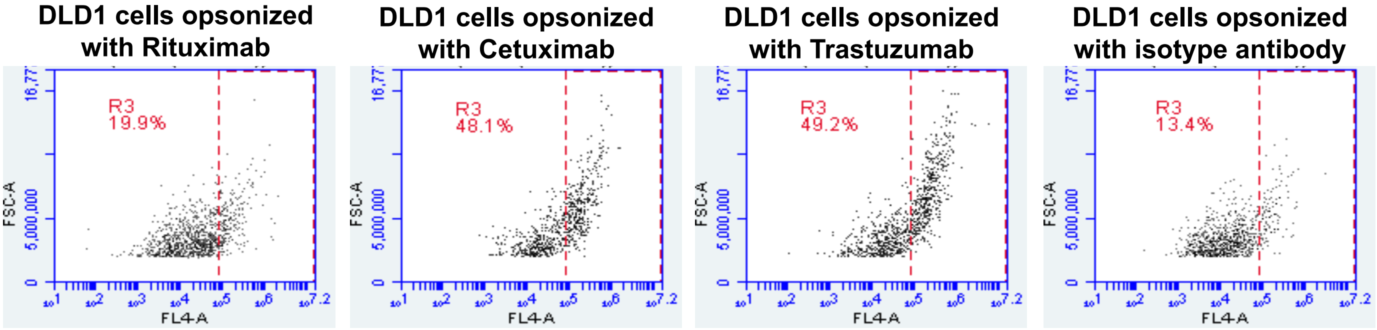

Supplementary Figure 16
Opsonin-dependent phagocytosis of DLD1 cells by engineered BMDMs. DLD1 cells were added to BMDMs and incubated at 37°C for 4 hours with indicated antibodies (rituximab (10 µg ml^-1), cetuximab (2 µg ml^-1), trastuzumab (2 µg ml^-1), or isotype IgG (2 µg ml^-1)).

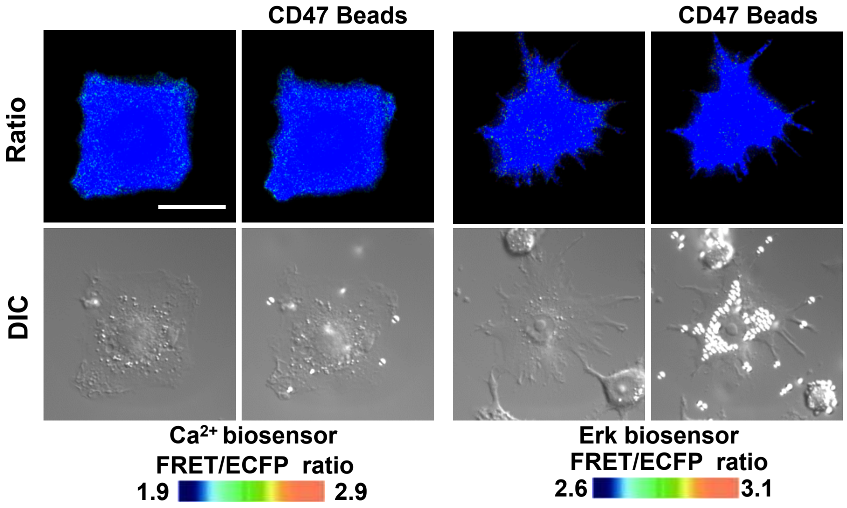

Supplementary Figure 17
Activation of downstream signals by SIRPα Syk-*i*SNAP depends on its kinase activity and fusion to SIRPα. Ratiometric and DIC images of RAW264.7 macrophages co-expressing the dark SIRPα Syk-*i*SNAP mutant with K402R in disrupting the Syk kinase domain together with Yc3.6 calcium biosensor or Erk biosensor were stimulated with CD47-coated beads. Color scale bar represent the FRET/CFP emission ratio, with cold and hot colors representing low and high FRET efficiency of the biosensors, respectively. Size scale bar, 20 μm.

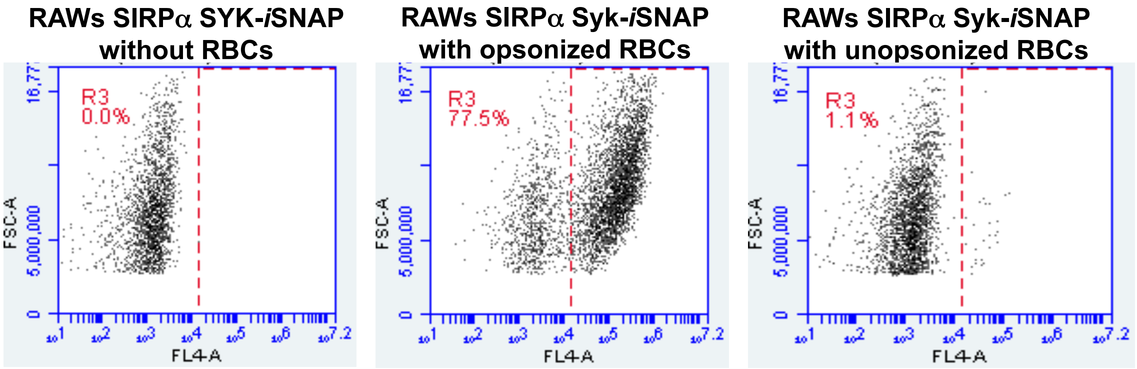

Supplementary Figure 18
Specificity of phagocytosis enhancing effect of SIRPa Syk-*i*SNAP. Opsonized or non-opsonized RBCs were incubated with RAW264.7 macrophages expressing SIRPa Syk-*i*SNAP. Ingested RBCs was quantified by flow cytometry. Numbers indicated percentage of macrophages with RBC engulfment in R3 gate.

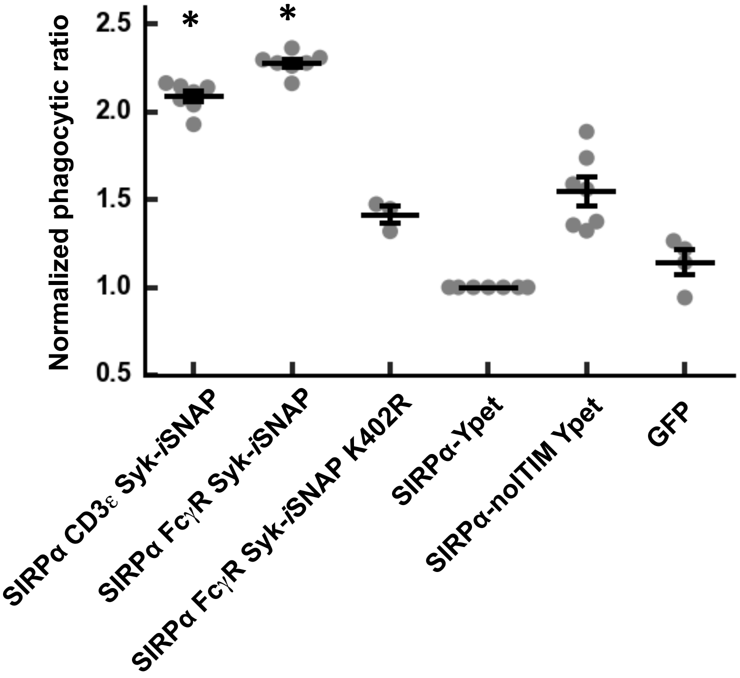

Supplementary Figure 19
Dot plot of normalized phagocytic ratio of RBCs by RAW264.7 macrophages expressing different constructs. Rabbit anti-human RBC IgG-opsonized RBCs were incubated at 37 °C for 30 min with RAW264.7 macrophages expressing SIRPα Syk-*i*SNAP with different BTAMs as indicated or control constructs (SIRPα CD3ε Syk-*i*SNAP indicates that the sensing peptide contains ITAM from CD3ε; SIRPα FcγR Syk-*i*SNAP: ITAM from FcγR; K402R: SIRPα Syk-*i*SNAP with a kinase dead mutation (K402R) in the Syk kinase domain; SIRPα: full length SIRPα fused with YPet; SIRPα-no ITIM: ITIM truncated SIRPα fused with YPet). Ingested RBCs was quantified by flow cytometry. Error bars represent s.e.m. (n= 7, 7, 3, 7, 7, 4), *: P<0.05.

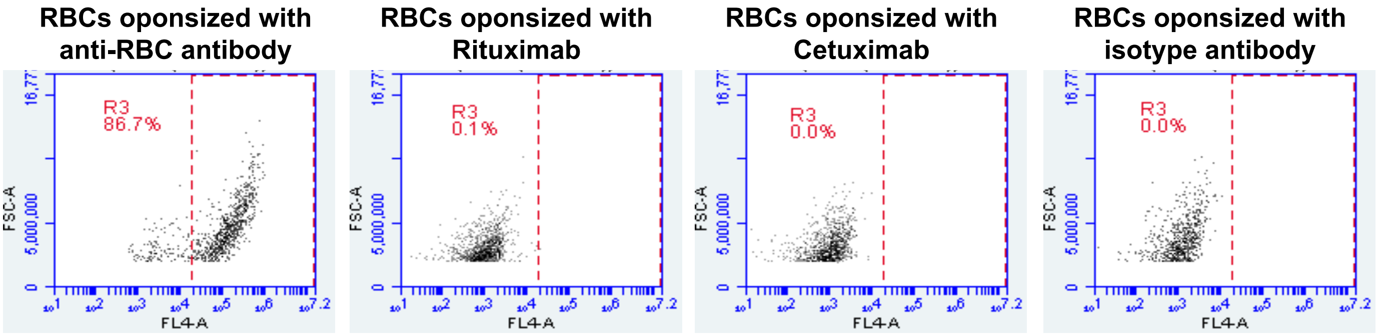

Supplementary Figure 20
Anti-TSA antibody-mediated phagocytosis of RBCs by engineered BMDMs is minimal. RBCs were added to BMDMs and incubated at 37°C for 4 hours with indicated antibodies (anti-RBC, rituximab, cetuximab, or isotype IgG).

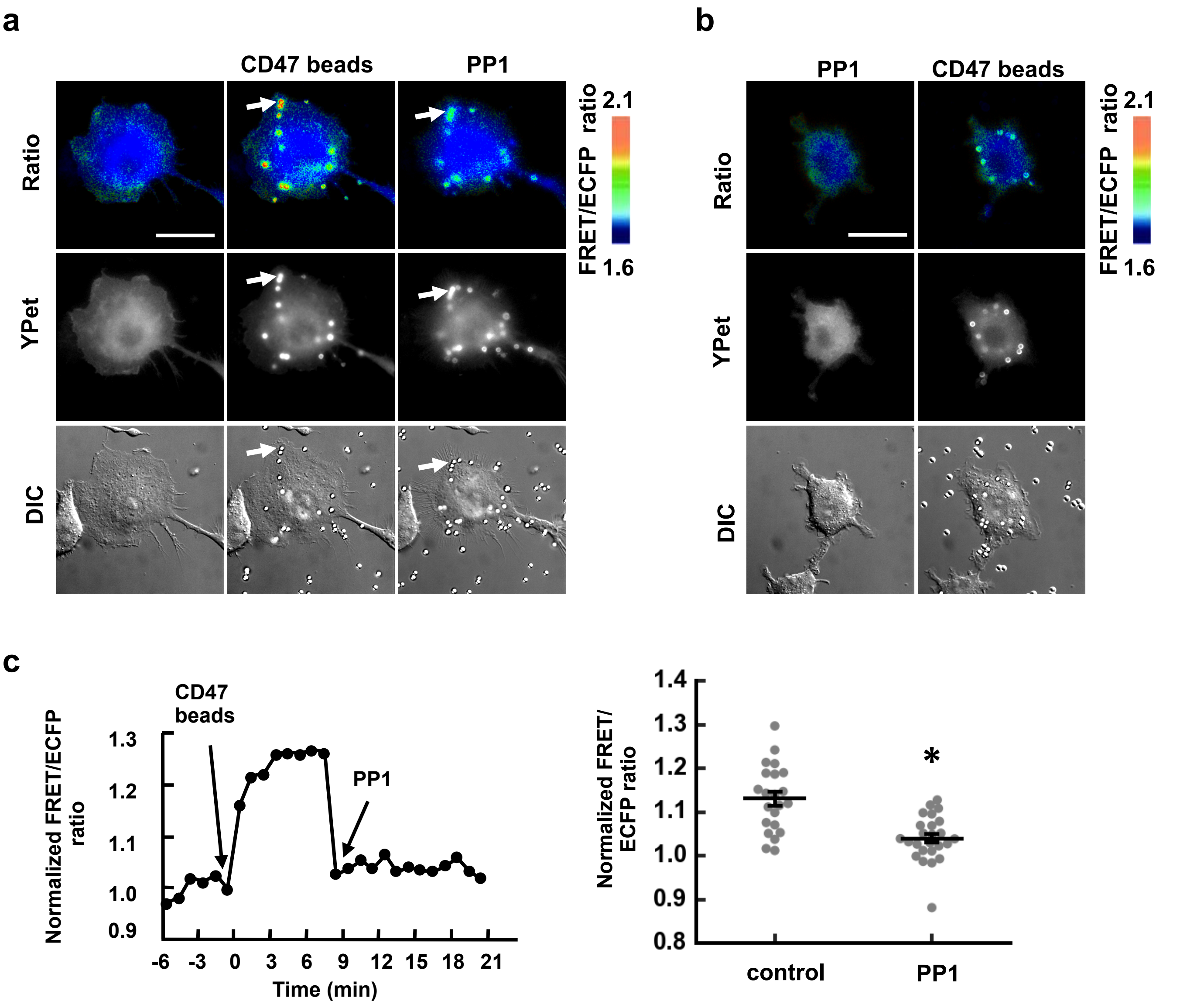

Supplementary Figure 21

SFKs mediated the CD47-induced activation of SIRPα Shp2-*i*SNAP. RAW264.7 macrophages were transfected with SIRPα Shp2-*i*SNAP. (**a**) Ratiometric, YPet and DIC images of RAW264.7 macrophages expressing SIRPα Shp2-*i*SNAP were stimulated with CD47 beads for 8 min before the Src inhibitor PP1 (10 μM) was added into the medium. Arrows point to the area where FRET/CFP ratio was collected for the time course in Figure 2D. (**b**) Ratiometric, YPet and DIC images of 10 μM PP1 pretreated RAW264.7 macrophages expressing SIRPα Shp2-*i*SNAP were stimulated with CD47-coated beads. (**C**) Left panel, the FRET ratio of SIRPα Shp2-*i*SNAP in response to CD47-coated beads and 10 μM SFKs inhibitor PP1 treatment in a representative macrophage. Right panel, dot plot of normalized FRET/ECFP ratio of SIRPα Shp2-*i*SNAP in response to stimulations by the CD47-coated beads with or without 10 μM PP1 pretreatment. (n=22, 22) *: $P<0.05$. Color scale bar represent the FRET/CFP emission ratio, with cold and hot colors representing low and high activities of reporter, respectively. Error bars represent s.e.m. Size scale bar, 20 μm.

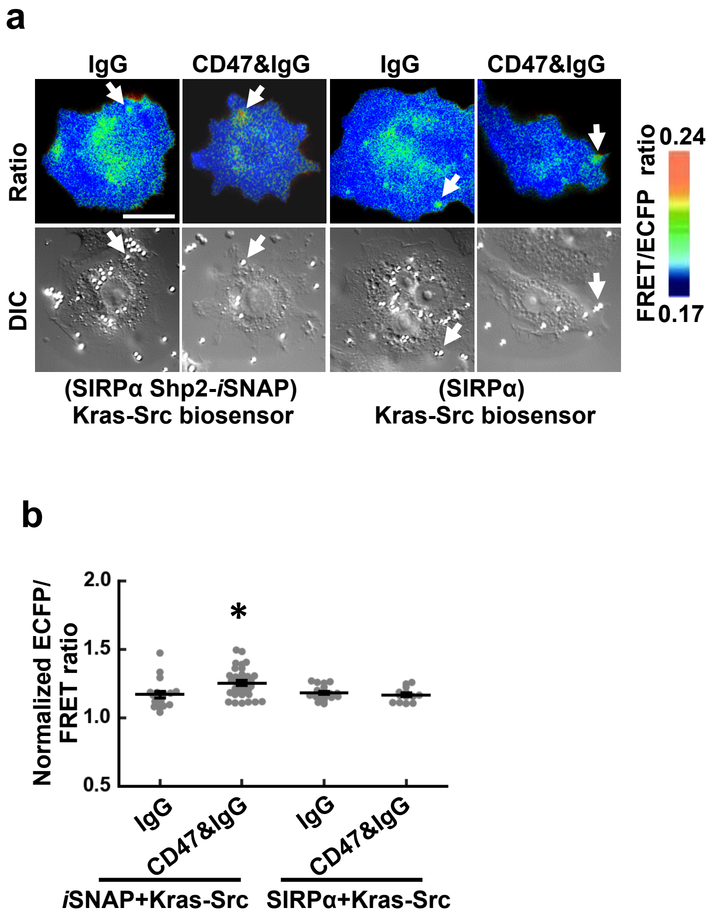

Supplementary Figure 22

(**a**) Ratiometric and DIC images of RAW264.7 macrophages expressing the dark SIRPα Shp2-*i*SNAP or control SIRPα-YPet together with the Kras-Src biosensor were stimulated with IgG or CD47 plus IgG beads. (**b**) Dot plot of normalized FRET/ECFP ratio of (**a**). (n= 19, 35, 20, 13) *: $P<0.05$. Color scale bar represent the FRET/CFP emission ratio, with cold and hot colors representing low and high FRET efficiency of the *i*SNAP, respectively. Error bars represent s.e.m. Size scale bar, 20 μm.

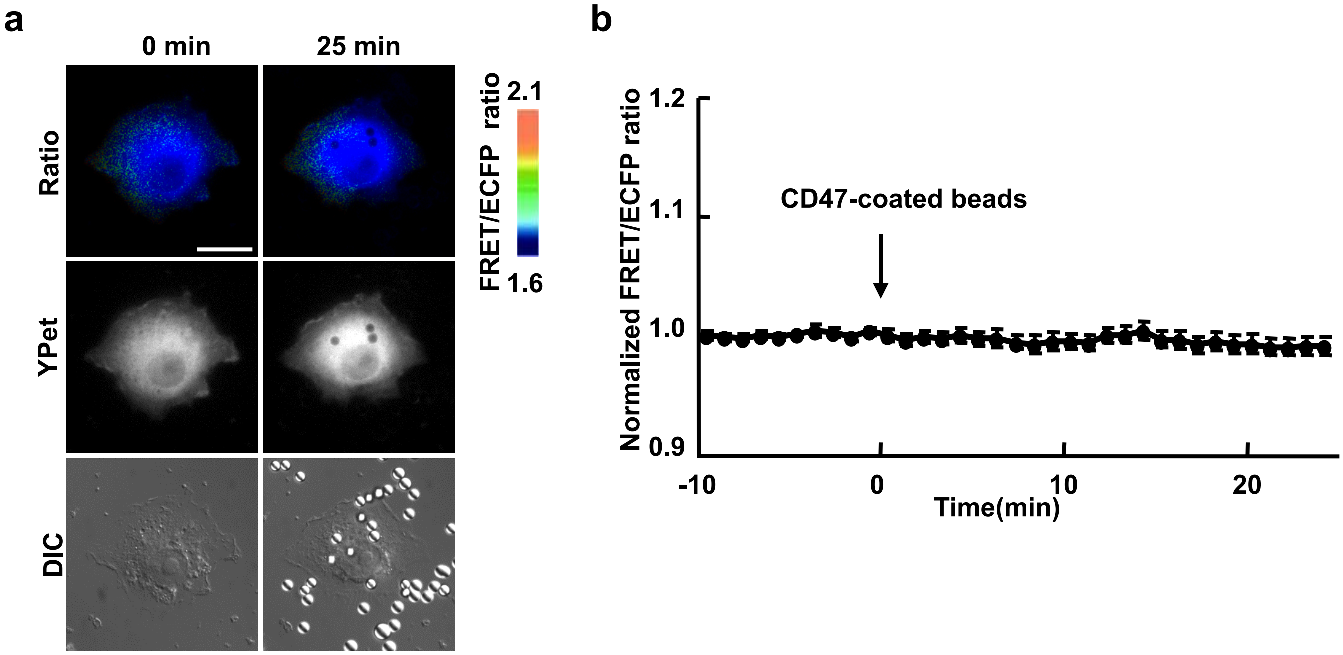

Supplementary Figure 23

Cytosolic Shp2-*i*SNAP did not respond to the engagement of CD47-coated beads. (**a**) Ratiometric, YPet, and DIC images and (**b**) time course of RAW264.7 macrophages expressing cytosolic Shp2-*i*SNAP stimulated with CD47-coated beads.

Construct Name	Substrate Sequence	Description	FRET increase	PTP increase
PDGFR 1009	SSVLYTAVQPNE	Y1009 and surrounding sequence from PDGFRβ (17)	Yes	No
2xPDGFR 1009	SSVLYTAVQPNEGSTSGSGKPGSGEGSTKGSTS GSGKPGSGEGSTKSSVLYTAVQPNE	Two Y1009 and surrounding sequence with linker (17)	Yes	No
IRS1	LNYIDLDLVKDGSTSGSGKPGSGEGSTKGSTSGS GKPGSGEGSTKLSTYASINFQK	Y1172 and Y1222 from IRS-1 with linker (18)	No	No
VIYFVP	SSVIYFVPQPNE	A selected sequence with high affinity to N-SH2 binding (19)	No	No
EIYEEF Src	EIYEEFQPNE	A selected sequence with high affinity for Src kinase (20)	No	No
Y542 Shp2	KGHEYTNIKYS	Y542 and surrounding sequence from Shp2 (21)	No	No
WMEY Src	WMEDYDYVHLQG	A selected sequence with high affinity for Src kinase (22)	No	No
BTAM	GGGGDITYADLNLPKGKKPAPQAAEPNNHTEY ASIQTS	bisphosphotyrosyl-containing activation motif from PTPNS1 (15)	Yes	Yes

Supplementary Table 1

The substrate sequences used in different *i*SNAPs and their FRET and PTP responses upon Src kinase phosphorylation *in vitro*. Phosphorylatable tyrosines are colored in red.
